# Successful Treatment of Plasma Cell-Rich Acute Rejection Using Bortezomib: A Case Report

**DOI:** 10.1155/2024/9226321

**Published:** 2024-09-06

**Authors:** Sara Hashemi, Reut Hod-Dvorai, Rebecca Tong, Liye Suo

**Affiliations:** ^1^ Division of Nephrology and Transplantation The State University of New York Upstate Medical University (SUNY Upstate Medical University), Syracuse, New York 13210, USA; ^2^ Department of Pathology The State University of New York Upstate Medical University (SUNY Upstate Medical University), Syracuse, New York 13210, USA

**Keywords:** allograft biopsy, bortezomib, plasma cell-rich acute rejection (PCAR), treatment

## Abstract

Plasma cell-rich acute rejection (PCAR), a relatively rare subtype of acute allograft rejection, is usually associated with a significantly lower treatment response rate and a higher graft failure rate. PCAR is characterized by the presence of more than 10% of plasma cells out of all graft infiltrating cells, with approximately 40%–60% of PCAR resulting in graft failure within a year. Currently, there is no gold standard for the effective treatment of PCAR. This case report demonstrates the potential treatment effect of bortezomib in PCAR. A 37-year-old woman with reflux nephropathy received a kidney transplant from a brain-dead kidney donor. The patient presented with an acute kidney injury with a serum creatinine level over 4 mg/dL 4 months after the surgery. The allograft biopsy showed acute T cell–mediated rejection (TCMR), Grade IIA, plasma cell-rich variant. There were diffuse polyclonal plasma cells infiltrating the renal parenchyma with marked tubulitis and focal endarteritis. She received a methylprednisolone pulse of 500 mg daily x3, followed by thymoglobulin (rATG) at 4.2 mg/kg. However, a repeated biopsy after 2 months showed persistent plasma cells infiltrate with increased interstitial fibrosis with tubular atrophy. Then, the patient was given one cycle of bortezomib with a total of four subcutaneous injections and continued immunosuppressants of tacrolimus, mycophenolate mofetil, and prednisone. Following the treatment, the patient's serum creatinine level trended down to 2 mg/dL, and a second repeat biopsy after 4 months showed a significant treatment effect with complete resolution of interstitial inflammation and decreased chronicity. Bortezomib is a proteasome inhibitor that prevents cell proliferation by inducing apoptosis in plasma cells and has shown great promise as a therapeutic agent for multiple myeloma. Our case suggests that bortezomib can also be used as a potential therapeutic intervention for patients with PCAR.

## 1. Introduction

Plasma cell-rich acute rejection (PCAR) in kidney transplantation, a relatively rare subtype of acute allograft rejection, is characterized by abundant mature plasma cells (at least 10% of the inflammatory cells) infiltrating the graft [1]. PCAR can be presented as all types of rejection including antibody-mediated rejection (AMR), T cell–mediated rejection (TCMR), and mixed rejection based on the Banff 2018 criteria [2]. Plasma-cell enrichment alone is an independent risk factor for a more severe adverse outcome and graft loss [3]. It is usually associated with a significantly less response rate to the standardized rejection treatment and a higher rate of graft failure, with approximately 40%–60% of PCAR resulting in graft failure within a year [4, 5]. Currently, there is no gold standard for the effective treatment of PCAR.

Bortezomib is a proteasome inhibitor that prevents cell proliferation by inducing apoptosis in plasma cells, and it has shown great promise as a therapeutic agent for multiple myeloma [6]. It has been experimentally applied to treat PCAR in a few published cases and has shown potential efficacy with 90% 2-year graft survival after rejection [7]. In addition, two cases with PCAR and AMR were successfully treated with bortezomib combined with plasma exchange, intravenous immunoglobulin, and rituximab. A marked decrease in the number of infiltrating plasma cells was confirmed by a posttreatment biopsy [8]. Here, we present a patient with the diagnosis of PCAR with TCMR and provide molecular and histologic evidence demonstrating the potential treatment effect of bortezomib in PCAR.

## 2. Case Presentation

**Figure 1:**
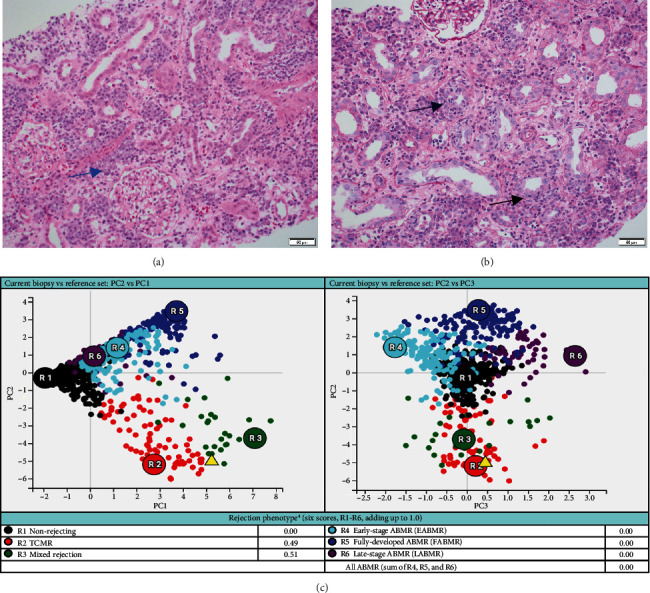
The histology and molecular findings from this patient's first biopsy showing severe plasma cell-rich rejection. (a, b) There are abundant plasma cells infiltrating (blue arrow in (a)) the renal cortex with marked tubulitis (black arrows in (b)) ((a) hematoxylin and eosin stain; (b) periodic acid-Schiff stain; magnification x200). (c) Rejection phenotype from the molecular microscopy diagnostic report showing severe T cell–mediated rejection.

A 37-year-old woman received a kidney transplant from a brain-dead kidney donor due to reflux nephropathy. The pretransplant calculated panel-reactive antibody (cPRA) was 0%, and the donor-recipient HLA mismatch was I A, I B, and II DR. The donor was positive for parvovirus B19, and the patient received the immunosuppressant induction of basiliximab and solumedrol with IVIG and the maintenance regimen with tacrolimus, solumedrol, and mycophenolate mofetil. The graft had immediate function and reached the baseline serum creatinine level of 1.0 mg/dL. However, the patient presented with acute kidney injury and serum creatinine over 4.0 mg/dL (0.6–1.1 mg/dL) 4 months after the transplant. Her plasma level of donor-derived cell-free DNA (dd-cfDNA) (Allosure, CareDx) was 4.8% (markedly elevated from the baseline level of <0.5%) (Table 1). Donor-specific antibodies (DSAs) were negative. Urine analysis showed proteinuria with a urine protein creatinine ratio (UPCR) of 0.34 mg/mg (normal range <0.15 mg/mg). Viral testings for polyomavirus and parvovirus B19 were negative. The allograft kidney biopsy showed acute TCMR, Grade IIA, plasma cell-rich variant (PCAR). There were diffuse and abundant polyclonal plasma cells infiltrating the renal parenchyma with marked tubulitis and focal endarteritis with mild interstitial fibrosis with tubular atrophy (Figure 1). No evidence of active AMR was present with negative C4d immunohistochemical staining. The corresponding molecular microscopy diagnostic report (MMDx, Thermo Fisher) showed severe TCMR with a rejection score of 0.87 (upper limit of normal is 0.1) and no AMR, and the atrophy-fibrosis score was 0.45 (upper limit of normal is 0.39).

**Figure 2:**
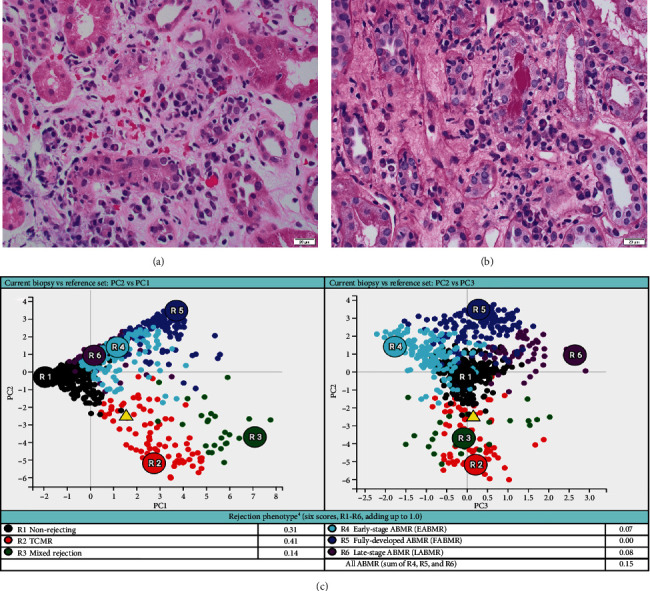
The histology and molecular findings from this patient's follow-up biopsy showing persistent plasma cell-rich rejection. (a, b) There are abundant plasma cells infiltrating the renal cortex with marked tubulitis ((a) hematoxylin and eosin stain; (b) periodic acid-Schiff stain; magnification x400). (c) Rejection phenotype from the molecular microscopy diagnostic report showing moderate T cell–mediated rejection.

She received methylprednisolone pulse 500 mg daily x3, followed by thymoglobulin (rATG) 1.5 mg/kg daily x4. Her serum creatinine trended down but stabilized around 2.8 mg/dL with a dd-cfDNA of 1.3%. A follow-up biopsy after 2 months demonstrated persistent plasma cell infiltration and marked tubulitis (Figure 2) with increased interstitial fibrosis with tubular atrophy. The corresponding MMDx showed moderate TCMR with a rejection score of 0.44 (upper limit of normal of 0.1) and no AMR, and the atrophy-fibrosis score was 0.70.

**Figure 3:**
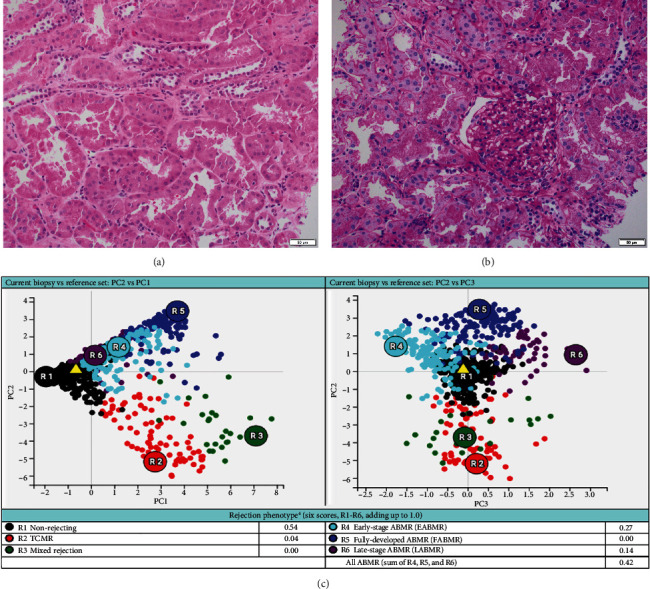
The histology and molecular findings from this patient's follow-up biopsy after bortezomib treatment showing no rejection. (a, b) There are normal-appearing renal cortex with no inflammation ((a) hematoxylin and eosin stain; (b) periodic acid-Schiff stain; magnification x200). (c) Rejection phenotype from the molecular microscopy diagnostic report showing no rejection.

Based on the poor response to the traditional TCMR treatment, the transplant nephrologist added bortezomib to target the plasma cell population. The patient was given one cycle of bortezomib at a dosage of 1.3 mg/m^2^ for a total of four subcutaneous injections and continued immunosuppressants of tacrolimus, mycophenolate mofetil, and prednisone. The patient's serum creatinine level gradually trended down to 2 mg/dL, with a significantly decreased dd-cfDNA level of 0.33%. Her UPCR was also decreased to 0.09 mg/mg. The second follow-up biopsy in 4 months showed a significant treatment effect with complete resolution of interstitial inflammation, no tubulitis, and markedly decreased chronicity (Figure 3). The corresponding MMDx showed no TCMR and no AMR, with a rejection score of 0.01, and the atrophy-fibrosis score was 0.31. During the treatment with bortezomib, no drug-related complications or active infections were reported.

## 3. Discussion

PCAR is a rare and poorly defined subtype of rejection in kidney transplantation. The development of abundant infiltrating mature plasma cells can be associated with immunosuppression noncompliance and certain infections [9]. To make an accurate diagnosis of PCAR, it is important to exclude the possibility of active infection (e.g., polyomavirus) and posttransplant lymphoproliferative disorder. Previous studies and case reports [10, 11] concluded that the plasma cell component in PCAR often poorly responds to conventional antirejection treatment and is independently associated with poor graft outcomes and survival rates. Antiplasma cell regimens including bortezomib have been proposed and experimentally applied in certain patients [12].

In this case report, the patient was initially treated with standard TCMR therapy following the diagnosis of PCAR and TCMR. Although there was a slight recovery of renal function with a decrease in serum creatinine level and lower rejection scores as measured by dd-cfDNA and MMDx (Table 1), there was persistent interstitial inflammation with marked tubulitis in histology and increased chronicity as evaluated by both histology and MMDx (Figure 2). After treatment with bortezomib, the patient returned to her baseline renal function along with a complete resolution of the inflammation and tubulitis in histology (Figure 3) and normal dd-cfDNA and MMDx. In addition, the previously increased interstitial fibrosis with tubular atrophy score was back to the patient's baseline level. This finding could be attributed to differences in biopsy sampling, but the other possibility is that bortezomib can reverse the chronic injury by depleting the infiltrating plasma cells. To our knowledge, this is the first case report showing histologic, molecular, and kidney function markers that were used to evaluate the treatment efficacy of bortezomib in a patient with PCAR. Our results suggest that bortezomib can be used as a potential and effective therapeutic intervention for PCAR.

Antiplasma cell medications other than bortezomib, such as the anti-CD38 monoclonal antibody felzartamab, have been tested in the treatment of rejection and showed treatment potential with acceptable safety profiles [13]. More studies are needed to investigate whether antiplasma cell regimens should be standardized as the treatment of choice for patients with PCAR.

## Figures and Tables

**Table 1:** Kidney function and molecular and urine markers at different times during the treatment.

**Markers**	**First biopsy**	**1st follow-up biopsy after traditional treatment**	**2nd follow-up biopsy after bortezomib therapy**
Serum creatinine (mg/dL)	4.0	2.8	2.0
dd-cfDNA (%)	4.8	1.3	0.33
MMDx rejection score	0.87	0.44	0.01
UPCR (mg/mg)	0.34	0.24	0.09

Abbreviations: dd-cfDNA, donor-derived cell-free DNA; MMDx, molecular microscopy diagnostic system; UPCR, urine protein creatinine ratio.

## Data Availability

The data that support the findings of this study are available from the corresponding author upon reasonable request.

## Nomenclature

AMR: antibody-mediated rejection
ATG: thymoglobulin
HLA: human leukocyte antigens
MMDx: molecular microscope diagnostic system
PCAR: plasma cell-rich acute rejection
TCMR: T cell–mediated rejection
UPCR: urine protein creatinine ratio
